# Views on HIV Disclosure Among Older Adults Living With HIV Who Are Childhood Sexual Abuse Survivors

**DOI:** 10.1093/geroni/igaa057.1088

**Published:** 2020-12-16

**Authors:** Monique Brown, Titilayo James, Chigozie Nkwonta, Amandeep Kaur

**Affiliations:** University of South Carolina, Columbia, South Carolina, United States

## Abstract

Rates of childhood sexual abuse (CSA) among people living with HIV are twice the CSA estimates among the general population. These statistics suggest that CSA prevalence may range from 16-22% among older adults living HIV (OALH). HIV disclosure continues to be a key consideration among people living with HIV. However, studies examining the views on HIV disclosure among OALH who are CSA survivors are lacking. Therefore, the aim of this study was to explore the views on HIV disclosure among OALH who are CSA survivors using a qualitative approach. Twenty-four adults aged 50-67 years, living with HIV and with a CSA history participated in the study. In-depth semi-structured interviews were conducted, audio-recorded and were analyzed using thematic analysis. The iterative analytic process included discussion of initial thoughts and key concepts, identification and reconciliation of codes, and naming of emergent themes. Three themes emerged: “You don’t have to tell the person if it’s just casual sex”, “Nothing ought to be hidden especially when you get ready to engage in sex”, and “As for me, I don’t disclose”. Views on HIV disclosure among OALH varied. Some participants stated that disclosure of HIV status should be dependent on the type of sexual relationship, while some OALH stated that participants should disclose regardless of type of relationship. Some participants were hesitant to disclose their HIV status due to anticipated HIV-related stigma. Understanding the perspectives on HIV disclosure among OALH with a CSA history may help to inform disclosure intervention programs for this vulnerable population.

